# Total body irradiation and iron chelation treatment are associated with pancreatic injury following pediatric hematopoietic stem cell transplantation

**DOI:** 10.18632/oncotarget.24646

**Published:** 2018-04-13

**Authors:** Natalia Maximova, Massimo Gregori, Roberto Simeone, Aurelio Sonzogni, Davide Zanon, Giulia Boz, Lorenzo D’Antiga

**Affiliations:** ^1^ Bone Marrow Transplant Unit, Institute for Maternal and Child Health-IRCCS Burlo Garofolo, Trieste, Italy; ^2^ Department of Pediatric Radiology, Institute for Maternal and Child Health-IRCCS Burlo Garofolo, Trieste, Italy; ^3^ Department of Transfusion Medicine, Trieste University Hospital, Trieste, Italy; ^4^ Department of Pathology, Hospital Papa Giovanni XXIII, Bergamo, Italy; ^5^ Pharmacy, Institute for Maternal and Child Health-IRCCS Burlo Garofolo, Trieste, Italy; ^6^ Department of Public Health, Clinical and Molecular Medicine, University of Cagliari, Cagliari, Italy; ^7^ Pediatric Hepatology, Gastroenterology and Transplantation, Hospital Papa Giovanni XXIII, Bergamo, Italy

**Keywords:** allogeneic HSCT, pediatric patients, acute pancreatic iron overload, exocrine pancreatic dysfunction, pancreatic shrinkage

## Abstract

Whereas many studies have addressed the risk of organ dysfunction following hematopoietic stem cell transplantation (HSCT), little is known about pancreatic susceptibility in this setting. We aimed to investigate the effect of iron overload (IO) and total body irradiation (TBI) on pancreatic function of children undergoing HSCT.

We retrospectively evaluated children admitted between 2012-2016 fulfilling the following criteria: normal pancreatic iron concentration (PIC), regular pancreatic function before HSCT, availability of abdominal magnetic resonance imaging with gradient-recalled-echo sequences and a full set of biochemical markers of IO and pancreatic function performed before HSCT and at discharge. We divided the patients according to the use of TBI or myeloablative chemotherapy (MCHT) in the conditioning regimen. All patients with severe IO or moderate IO with a high risk of engraftment delay or transplantation-related complications underwent chelation therapy with deferoxamine (DFO) from the first day of conditioning to discharge.

63 patients had a HSCT in the study period, 13 did not fulfill the inclusion criteria; 50 (25 in each group) are included in the analysis, and did not show differences at baseline evaluation. At follow up testing the TBI group showed a significantly higher PIC (107,8±100,3 μmol/g vs 28,4±37,9 in MCHT group, p<0,0001). In the TBI group the patients who had DFO treatment had higher PIC (223,2±48,8 μmol/g vs 55,7±10,5 without DFO treatment, p<0,0001), and all patients having PIC >100 μmol/g at follow up had DFO-based chelation therapy, versus 26% of those with lower PIC (p<0,0001). The number of patients presenting exocrine pancreatic dysfunctions one month after transplantation was significantly higher in the TBI group (48% vs 4%; p<0.0001). The mean pancreatic volume reduction was significantly greater in the TBI group (39,1% vs 0,9% in the MCHT group; p<0,05), and was significantly worse on those who received DFO therapy.

Based on our data, we suggest that TBI is detrimental for pancreatic functions, and speculate that DFO may contribute to the rapid pancreatic IO observed in these patients.

## INTRODUCTION

In recent years, allogeneic hematopoietic stem cell transplantation (HSCT) has become an important technique to treat pediatric diseases including hematological and oncological diseases as well as inborn errors of metabolism. Iron overload (IO) is associated with poor prognoses of patients who undergo allogeneic HSCT for hemato-oncological diseases, but it appears to be an underestimated transplantation-related complication [1]. IO correlates with an increased risk of non-relapse-related mortality following HSCT and may enhance the risk of acute and chronic graft-versus-host disease (GVHD) [2–4], infections, sinusoidal obstruction syndrome, idiopathic pneumonia syndrome, and chronic liver dysfunction during the post-transplantation period [5].

Iron is an essential element in the body, and its storage is finely regulated. However, humans lack an excretory pathway for excess iron [6]. Therefore IO can persist for years after HSCT, and its toxic effects might play a role in the development of long-term transplantation-related complications. Exposure to transfused red blood cells, both during treatment of the underlying disease and the post-transplantation period, is considered as the main cause of IO in HSCT recipients. However, excessive iron storage in patients with hematological malignancies is considered to be multifactorial. Intensive cytotoxic therapy before HSCT causes bone marrow and neoplastic cell lysis, releasing free and protein-bound iron [7, 8].

Although hepatic and cardiac IO have been studied extensively, limited data are available on IO in other organs. Iron metabolism varies in different organs. The pancreas selectively takes up non-transferrin-bound iron (NTBI) [9]. Pancreatic IO is quite frequent among patients with transfusion-dependent anemias [10]. The percentage of pancreatic siderosis in this setting of patients reaches 80% [11, 12]. Nevertheless, no data about the prevalence of pancreatic IO in patients who undergo HSCT for malignancies or genetic disorders are available in the literature.

The pathophysiology of systemic IO in iron-loading anemias, such as β-thalassemia and myelodysplastic syndromes, has been widely studied. Iron within transfused erythrocytes preferentially deposits in reticuloendothelial cells of the liver, spleen, and bone marrow. Iron storage capacity of the reticuloendothelial system of an adult is about 10 g [13]. One unit of PRBCs provides an increase of about 200–250 mg to the total body iron pool, and clinical evidence has shown that IO can occur after transfusion of up to 20 PRBC units [5]. IO in patients undergoing HSCT for hematological malignancies results from cytotoxic therapy for primary diseases and conditioning before HSCT, in addition to pre-transplantation blood transfusions. Myeloablative conditioning destroys bone marrow and cancer cells, and damages hepatic cells, releasing intracellular iron pools and increasing free iron levels [7, 8].

The causes and potential effects of pancreatic IO on transplant outcomes have not been determined. Our HSCT protocol includes the use of magnetic resonance imaging (MRI) with various gradient-recalled-echo sequences to quantitatively measure iron concentrations in abdominal parenchymal organs of pediatric patients before and after allogeneic HSCT [14].

In this study, we retrospectively evaluated the association between pancreatic IO and the conditioning regimen, pre-transplantation liver iron concentration, and chelation therapy in children who had been admitted to our Transplant Unit over the past 5 years. Moreover, we focused on correlations between pancreatic IO and clinical outcomes.

## RESULTS

Sixty-three consecutive pediatric patients had undergone allogeneic HSCT in our center over the past 5 years. Among them, six children were excluded because they did not undergo pre-transplantation MRI evaluation of iron concentrations, six were excluded because they did not suffer from hematological or oncological diseases and did not exhibit IO before transplantation, and one patient was excluded because he presented with pancreatic IO before transplantation. The study group was represented by the remaining 50 patients aged under 18 years with pre-transplantation IO involving at least one of the examined organs, a normal pancreatic iron concentration, and regular pancreatic functions. This cohort of 50 patients was divided into two groups based on the conditioning regimen: myeloablative chemotherapy (MCHT; 25 patients) and TBI (25 patients). Baseline patients characteristics are listed in Table 1.

**Table 1:** Baseline characteristics at the time of transplantation

Pre-transplant baseline characteristics	Whole cohort
**Number of patients (%)**	50 (100)
**Gender**	
Male (%)	32 (64,0)
Female (%)	18 (36,0)
**Age at transplant, years, mean (± SD)**	8,45 (4,8)
**Underlying disease, number (%):**	
Acute lymphoblastic leukemia	24 (48,0)
Acute myeloid leukemia	8 (16,0)
Myelodysplastic syndrome	12 (24,0)
Hemoglobinopathy	4 (8,0)
Solid tumor	2 (4,0)
Disease stage, number (%):^*^	
Early	18 (46,2)
Intermediate	14 (28,2)
Late	12 (25,6)
**Myeloablative conditioning, number (%):**	
MCHT-based	25 (50,0)
TBI-based	25 (50,0)
**Iron overload, number (%):**	
Mild	21 (42,0)
Moderate	10 (20,0)
Severe	19 (38,0)
**DFO-chelation therapy, number (%):**	22 (44,0)
**Comorbidity, number (%):**	39 (78,0)
Hepatobiliary	24 (48,0)
Renal disease	1 (2,0)
Metabolic syndrome	7 (14,0)
Infection	7 (14,0)

MCHT, myeloablative chemotherapy; TBI, total body irradiation; SD, standard deviation; DFO, deferoxamine.

^*^Disease stage was defined according to previously published classification.

This classification is applied to patients with acute leukemia and MDS only [31].

### Pre-transplantation characteristics

Pre-transplantation characteristics of the patients are shown in Table 2. There were no statistically significant differences in the number or age of patients between the two groups (p>0.05). We analyzed pre-transplantation renal and hepatic functions in both groups. In the cohort, only one patient in the TBI group had chronic mild renal insufficiency with an eGFR from 60 to 90 ml/min/1.73 m^2^. The most common cause of hepatic injury was severe IO in both groups: 18% and 20% MCTH and TBI groups, respectively. The percentage of patients showing drug-induced liver toxicity was greater in the TBI group (12% vs 2% in the MCHT group). Nevertheless, no statistically significant difference was found. Moreover, no significant differences in the incidences of liver injury, viral or autoimmune hepatitis, steatosis, or siderosis were found between the two groups. The total number of packed red blood cell (PRBC) units received by the patients before HSCT in the two groups was not significantly different. The grade of siderosis, expressed as MTIC or calculated in each organ (LIC, PIC, SIC, and BIC) was not statistically different.

**Table 2:** Pre-transplant characteristics of the four study groups

BASELINE CHARACTERISTICS	MCHT GROUP	TBI GROUP	*P –*value^*^
**Number of patients (% of whole cohort)**	25 (50,0)	25 (50,0)	NS
**Age, years, mean (± SD)**	7,4 (6,1)	9,6 (4,6)	NS
**Renal disease, number (%)^#^**	0	0	-
**eGFR, ml/min/1,73m^2^, mean (± SD)**	228,9 (99,0)	195,0 (91,6)	NS
**Liver injury, total number (%):**	11 (22,0)	13 (26,0)	NS
- viral hepatitis, number (%)	2 (4,0)	1 (2,0)	NS
- autoimmune hepatitis, number (%)	2 (4,0)	0	NS
- steatosis, number (%)	4 (8,0)	6 (12,0)	NS
- siderosis, number (%)^§^	9 (18,0)	10 (20,0)	NS
- drug toxicity, number (%)	1 (2,0)	6 (12,0)	NS
**PRBC units received, mean (± SD)**	17,7 (18,9)	20,4 (15,5)	NS
**MTIC, grade, mean (± SD):**	1,6 (0,7)	1,9 (0,7)	NS
**MTIC, μmol/g, mean (± SD):**	113,1 (58,2)	144,4 (66,3)	NS
- liver iron concentration, mean (± SD)	127,0 (86,7)	71,0 (95,0)	NS
- pancreas iron concentration, mean (± SD)	22,7 (12,1)	27,2 (12,1)	NS
- spleen iron concentration, mean (± SD)	135,4 (97,8)	174,2 (109,2)	NS
- bone iron concentration, mean (± SD)	166,4 (83,4)	205,0 (70,8)	NS

MCHT, myeloablative chemotherapy; TBI, total body irradiation; SD, standard deviation; eGFR, estimated

glomerular filtration rate; PRBC unit, packed red blood cell unit; MTIC, mean tissue iron concentration.

NS: not significant (p>0.05).

^#^Patients with eGFR < 90 ml/min/1,73m^2^.

^§^Only histological diagnosis of severe siderosis with initial fibrosis.

^*^P-value from analysis of variance or chi-square when appropriated.

### Transplantation-related features at baseline and 1 month after HSCT

MRI examination was performed at 32.8±2.6 days after transplantation in the MCHT group and at 33±2.8 days in the TBI group. MTIC, LIC, SIC, and BIC were not statistically different between the two groups. Conversely, PIC was significantly higher in the TBI group (107.8±100.3 vs 28.4±37.9 μmol/g in the MCHT group, p<0.0001). Furthermore, pre- and post-transplantation PIC values were compared. In the MCHT group, the PIC value remained unchanged (22.7±12.1 vs 28.4±37.9 μmol/g, p>0.05), whereas PIC was significantly increased after transplantation in the TBI group (27.2±12.1 vs 107.8±100.3 μmol/g, p<0.0001). In the TBI group the patients who had DFO treatment had higher PIC (223,2±48,8 μmol/g vs 55,7±10,5 without DFO treatment, p<0,0001). The incidence of transplantation-related liver complications, such as sinusoidal obstruction syndrome, GVHD, infections, and conditioning drug toxicity, was similar in the two groups. A significant difference was found in eGFR values of the two groups (p<0.005). The TBI group had lower values. The number of patients with exocrine pancreatic dysfunctions (low serum amylase and lipase levels and a low fecal elastase-1 concentration) at 1 month after transplantation was significantly higher in the TBI group (48% vs 4% in the MCHT group, p<0.0001). No patient developed endocrine pancreatic dysfunctions.

We found no difference in the number of PRBC units received by patients of the two groups during their stay at the Transplant Unit. Evaluated post-transplantation characteristics of patients are shown in Table 3.

**Table 3:** Transplant-related features: differences between two groups one month after transplantation

VARIABLES	MCHT GROUP	TBI GROUP	*P*- value^*^
**Number of patients (% of whole cohort)**	25 (50,0)	25 (50,0)	NS
**Pancreatic dysfunction, number (%):**			
- exocrine	2 (4,0)	24 (48,0)	<0,0001
- endocrine	0	0	-
**Liver injury, total number (%):**	11 (22,0)	11 (22,0)	NS
- SOS, number (%)	3 (6,0)	1 (2,0)	NS
- GVHD, number (%) ^+^	5 (10,0)	2 (4,0)	NS
- infections, number (%)	3 (6,0)	4 (8,0)	NS
- drug toxicity, number (%)	2 (4,0)	5 (10,0)	NS
**eGFR, ml/min/1,73m^2^, mean (± SD)**	188,4 (72,7)	139,5 (54,5)	<0,05
**PRBC units received, mean (± SD)^#^**	4,4 (2,1)	3,5 (1,8)	NS
**MTIC, grade, mean (± SD)**	1,9 (0,8)	2,2 (0,8)	NS
**MTIC, μmol/g, mean (± SD):**	138,4 (63,0)	177,7 (76,3)	NS
- Liver iron concentration, mean (± SD)	153,2 (80,5)	182,2 (93,6)	NS
- Pancreas iron concentration, mean (± SD)	28,4 (37,9)	107,8 (100,3)	<0,0001
- Spleen iron concentration, mean (± SD)	177,4 (104,3)	201,0 (103,1)	NS
- Bone iron concentration, mean (± SD)	191,0 (69,5)	219,6 (50,4)	NS
**Deferoxamine treatment, total number (%)**	11 (44)	11 (44)	NS
**Mean pancreatic volume reduction, (%)^α^**	0,6	39,1	<0,0001

MCHT, myeloablative chemotherapy; TBI, total body irradiation; SD, standard deviation; VOD, sinusoidal obstruction syndrome; GVHD, graft versus host disease; eGFR, estimated glomerular filtration rate; PRBC unit, packed red blood cell unit; MTIC, mean tissue iron concentration.

NS: not significant (p>0.05).

^+^Only hepatic GVHD ≥ grade 2, according to clinical criteria.

^#^Only transfusion received during the stay at the Transplant Unit.

^α^Percentage difference between baseline mean pancreatic volume and mean post-transplant pancreatic volume.

^*^P-value from analysis of Mann Whitney test, Fisher's test or chi-square when appropriated.

### Factors associated with pancreatic IO

The cohort of 50 patients was divided into two groups based on post-transplantation PIC values (cut-off threshold: 100 μmol/g) to identify the causes of pancreatic IO in the first month after HSCT. The type of conditioning, mean body baseline iron store, and deferoxamine (DFO) chelation therapy were considered. The percentage of patients who underwent TBI-based conditioning was significantly higher in the high PIC group [11 patients (91.6%) vs one patient (8.3%) in the MCHT group, p<0.0001]). As expected, baseline MTIC in the high PIC group was almost three times higher (p<0.0001). All patients in the high PIC group underwent DFO-based chelation therapy, whereas only 26% of patients in the normal PIC group did (p<0.0001). Unexpectedly, all patients of the high PIC group had developed acute pancreatic IO during DFO chelation therapy. PICs increased from a mean baseline value of 30.8±14.1 μmol/g to 211.7±46.7 μmol/g within 1 month after transplantation (p<0.0001), while the iron concentration did not increase in the other examined organs.

### Factors associated with pancreatic volume modifications

**Figure 1:**
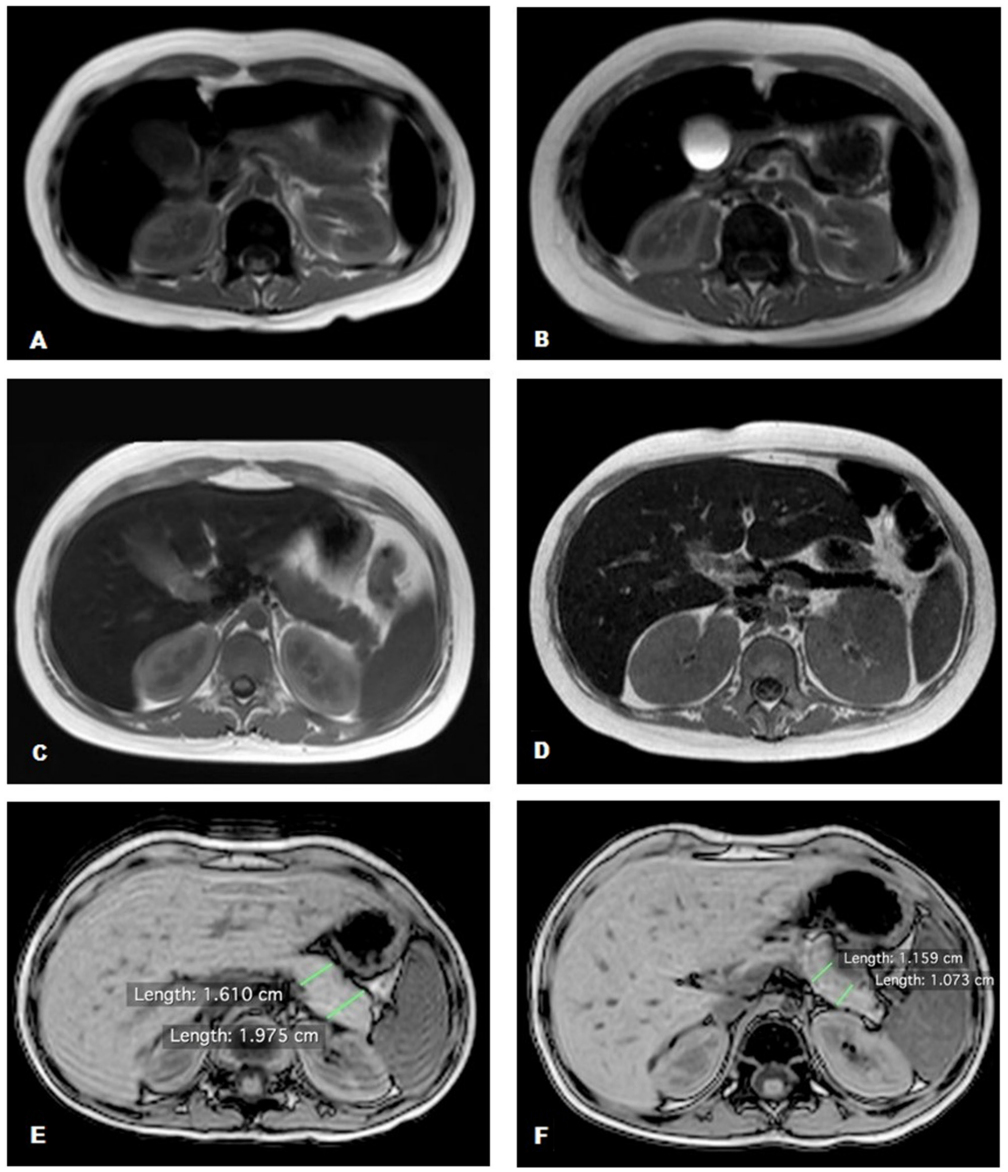
Abdominal MRI T2 fast field echo sequences **(A, C, E)** Normal pancreas signal and volume; **(B, D)** hypointense pancreas signal with reduction of pancreatic volume; **(F)** normal pancreas signal with a reduction of pancreatic volume.

Baseline and post-transplantation pancreatic volumes were compared in the two groups. The mean pancreatic volume reduction was significantly higher in the TBI group (39.1% vs 0.9% in the MCHT group; p<0.05). Patients in the TBI group, who underwent DFO chelation therapy, had the highest mean volume reduction from 31.8 cm^3^ to 16.2 cm^3^, corresponding to a mean reduction of 49.2% (p<0.01) with a maximum reduction of 61.9% and minimum of 36.3%. Patients in the group without DFO chelation therapy had a lower mean volume reduction from 30.2 cm^3^ to 20.9 cm^3^, corresponding to a mean reduction of 31.2% (p<0.01) with a maximum reduction of 40.1% and minimum of 19.3%. The difference in the decrease of pancreatic volume between the two groups was statistically significant (p<0.01). Images of the two most impressive cases of acute pancreatic IO with reduction of pancreatic volume are shown in Figure 1A–1D.

To assess the role of TBI in pancreatic shrinkage, we scheduled an MRI scan at 48–72 hours after the last TBI session for the last three patients treated. All patients had a significant pancreatic volume reduction. The first patient had a reduction of 30.2% (20.9 cm^3^vs 14.6 cm^3^ baseline volume at 48 hours after TBI), the second patient had a reduction of 36.6% (21.3 cm^3^ vs 13.5 cm^3^ baseline volume at 72 hours after-TBI), and the third patient had a reduction of 35.4% (36.2 cm^3^ vs 23.4 cm^3^ baseline volume at 72 hours after TBI). The PICs of all three patients remained unchanged in the first 48–72 hours after TBI (Figure 1E, 1F).

**Figure 2:**
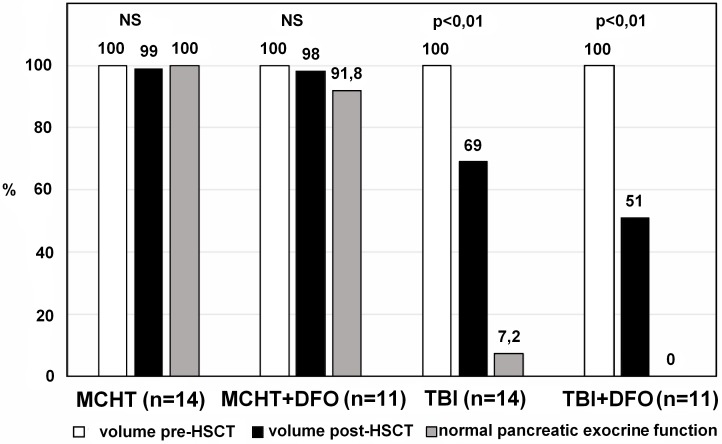
Significant reductions of pancreatic volumes and pancreatic exocrine functions in the TBI-based group compared with almost unchanged pancreatic volumes and functions in the MCHT-based group MCHT, myeloablative chemotherapy; DFO, deferoxamine; TBI, total body irradiation; HSCT, hematopoietic stem cell transplantation.

Correlations were evaluated between pancreatic endocrine and exocrine functions and acute pancreatic IO. Endocrine functions, expressed as secretion of insulin, c-peptide, and hemoglobin A1c, remained unchanged after transplantation in both groups. Conversely, exocrine functions were impaired in all 12 patients with acute pancreatic IO (Table 4; Figure 2).

**Table 4:** Features associated with pancreatic iron overload in the first month after HSCT

VARIABLES	PIC < 100 μmol/g	PIC > 100 μmol/g	*P*-value^*^
**Number of patients (% of whole cohort)**	38 (76)	12 (24)	
**MCHT-Based Conditioning (%)**	24 (63,1)	1 (8,3)	<0,05
**TBI-Based Conditioning (%)**	14 (36,8)	11 (91,6)	<0,05
**Pre-Trasplant MTIC, μmol/g, mean (±SD)**	108,4 (56,8)	192,9 (36,1)	<0,0001
**Post-Transplant MTIC, μmol/g, mean (±SD)**	132,2 (58,1)	239,8 (46,8)	<0,0001
**Pre-Trasplant PIC μmol/g, mean (±SD)**	23,6 (11,6)	30,8 (14,1)	NS
**Post-Trasplant PIC μmol/g, mean (±SD)**	22,8 (12,0)	211,7 (46,7)	<0,0001
**Pre-Trasplant Pancreatic volume, mean (±SD)**	28,2 (11,6)	32,0 (9,9)	NS
**Post-Trasplant Pancreatic volume, mean (±SD)**	24,6 (11,2)	17,7 (8,3)	<0,05
**Pancreatic Exocrine Function:**			
- Serum amylase, U/l, mean (± SD)	16,7 (10,5)	4,3 (2,6)	<0,0001
- Serum lipase, U/l, mean (± SD)	18,8 (11,2)	6,0 (2,1)	<0,0001
- Fecal elastase, ug/g, mean (± SD)	418,9 (139)	110,6 (125,6)	<0,0001
**Pancreatic Endocrine Function**			
- Insulin, μUI/mL, mean (± SD)	39 (19,8)	49,7 (23,4)	NS
- C-peptide, ng/mL, mean (± SD)	4,8 (3,6)	6,3 (3,3)	NS
- Hemoglobin A1c, mmol/mol, mean (± SD)	35,6 (5,2)	38,2 (5,6)	NS
**DFO Chelation Therapy, number of patients (%)**	10 (26,3)	12 (100)	<0,0001

PIC, pancreatic iron concentration; MCHT, myeloablative chemotherapy; TBI, total body irradiation; SD, standard deviation; MTIC, mean tissue iron concentration; DFO, deferoxamine.

NS: not significant (p>0.05).

^*^P-value from analysis of Mann Whitney test or Fisher's test when appropriated.

## DISCUSSION

According to the pediatric international protocols, different types of MCHT-based conditioning are used in patients affected by all types of leukemia, except for acute lymphoblastic leukemia in children older than 2 years, and by all those pathologies that include an HSCT in the therapeutic protocol (myelodysplastic syndrome, hemoglobinopathies, aplastic anemia, inborn errors of metabolism and primary immunodeficiencies). On the other hand, the most common myeloablative conditioning regimens for acute lymphoblastic leukemia in pediatric patients is based on TBI. Radiosensitivity has been accurately estimated in the liver, kidneys, and gastrointestinal tract [15–18]. However, no systematic studies have provided data about the radiosensitivity of human pancreatic tissue or radiation-induced pancreatic insufficiency. The strong structural similarity between pancreatic and salivary glands suggests that radiation-associated injury of the pancreas may be similar to that of the salivary gland [19]. The high radiation sensitivity of salivary tissue leads to reproductive death of acinar progenitor cells, even though secretory cells in salivary glands have a slow turnover [20].

Knowledge about radiation-induced damage of the exocrine pancreas is based on autopsy findings and experimental animal studies. Acinar cell necrosis, duct cell injury, atrophy of ductules, vascular lesions, and diffuse fibrosis have been observed in the pancreas many years after radiotherapy [21]. However, little is known about acute radiation-induced pancreatic injury. Wydmanski et al. reported that gastric cancer patients subjected to chemo-radiotherapy develop hypolipidemia more frequently than diabetes. These data show that acinar cells are more radiosensitive than β-cells [19]. Our data confirmed that acute pancreatic injury due to TBI affects exocrine functions only. All patients who underwent TBI before HSCT had normal baseline exocrine and endocrine activities. Twenty-four out of 25 (96%) TBI-treated patients developed mild exocrine insufficiency within 1 month after HSCT. Endocrine functions remained normal in all 25 patients. In the MCHT-treated group, the incidence of exocrine pancreatic insufficiency was clearly lower. Only two out of 25 (8%) patients in this group developed exocrine insufficiency. In the TBI group, no patients developed endocrine pancreatic insufficiency. In addition to the significant loss of pancreatic volume (mean volume reductions of 31.2% and 49.2% in TBI and TBI-DFO groups, respectively) after TBI-based conditioning, these data suggest that pancreatic tissue is highly sensitive to radiation.

By studying the last three patients with early MRI follow-up, we found that pancreatic gland loses of more than one third of its basal volume had already occurred at 48–72 hours after the last TBI session. In addition to the structural affinity between pancreatic and salivary glands, the anatomical location occupied by the pancreas exposes it directly to γ-rays during TBI sessions. The only protected part of the pancreas is its head, which is covered by the liver, while the body and tail are only covered by the stomach that is usually empty because of pre-procedure fasting.

Our study suggests another reason. Patients treated with DFO had more severe and rapid iron storage compared with those who did not receive DFO, suggesting a synergic role of TBI and DFO in the severity of pancreatic iron deposition. To explain this synergic toxic effect, we hypothesized an interaction of several factors.

Increased total body iron load causes a rise in toxic forms of iron such as NTBI, labile plasma iron, and labile cellular iron (ferrous iron, Fe^2+^). After a TBI-based conditioning regimen, transferrin saturation exceeds 80% and levels of NTBI rise [22]. When non-transferrin-bound iron levels are high, serious oxidant damage may occur, especially in the presence of oxidants produced during acute tissue inflammation such as during TBI [23, 24]. Oxidative damage *in vivo* is often ascribed to the Fenton reaction in which Fe^2+^ reacts with hydrogen peroxide (H_2_O_2_) to produce ferric iron (Fe^3+^, ferrioxamine), hydroxide anions (OH^-^), and highly reactive hydroxyl radicals (OH^·^) [25]. The radicals produced in the Fenton reaction draw hydrogen from polyunsaturated fatty acids in the cell membrane, inducing non-enzymatic lipid peroxidation. Radical compounds give rise to new radicals, producing a chain reaction [26].

During TBI, the main immediate consequence of the absorption of high energy radiation is the production of free radicals. In the presence of oxygen, these radicals may lead to toxic reactions [27]. Because of its endocrine secretion, the pancreatic gland is widely vascularized and thus richly oxygenated. We assume that, owing to its rich oxygenation, radiation-induced damage leads to the production of a greater amount of free radicals in pancreatic tissue compared with other abdominal organs. Therefore, we consider that pancreatic oxidative stress may derive from the combined effects of TBI and systemic IO. In addition, a third factor should be considered. DFO is a powerful chelator of ferrioxamine, which is able to quench oxidizing reactions and is itself a capable radical scavenger. During the Fenton reaction, it effectively inhibits iron ion-dependent lipid peroxidation and the generation of highly reactive oxidizing species. *in vitro* models have shown that DFO inhibits the Fenton reaction because of its ability to scavenge OH^·^ and H_2_O_2_, and to a lesser extent O_2_^·^ (superoxide radicals), and forms water soluble complexes with ferrioxamine [28]. Most likely, it is impossible to reproduce this *in vitro* model in an organism with heavy IO. We considered that DFO is attracted to the pancreas by the enormous amount of free radicals produced after irradiation, but as it circulates in an environment full of iron, it probably arrives at the pancreas already bound to ferrioxamine.

Ferrioxamine is a coordination complex in which bonds between the coordination center (ferrioxamine) and the complexing agent (DFO) are much weaker than those between DFO and free radicals. In the case of excess of OH^·^ and H_2_O_2_, DFO creates a very stable bond with them to release ferrioxamine. Ferrioxamine released by DFO joins the Fenton-catalyzed Haber-Weiss reaction [29]. As a result, excess ferrioxamine is produced, which is partly released by cells damaged directly by γ-rays and generated by the Haber-Weiss reaction, in addition to the high levels of NTBI and the labile iron pool because of the severe systemic iron accumulation.

NTBI is avidly taken up by several tissues, especially the liver. The liver and spleen are already completely overloaded with iron. Therefore, the pancreas is the only non-saturated organ available, which could be a suitable scenario to explain the acute pancreatic IO after TBI-based conditioning in patients who underwent chelation therapy with DFO.

Our study has several limitations. First, it is a retrospective study. However, to the best of our knowledge, this is the first report of development of acute IO during chelation therapy. Unfortunately, our study does not provide any material evidence supporting the hypothesis about the involved mechanism. The pancreas is an organ that cannot be biopsied unless there is a large neoformation. Therefore, it is only possible to establish with certainty the mechanism by which iron saturates pancreatic tissue in the presence of DFO using an animal model.

We also need a longer follow-up period to show that our patients do not develop long-term clinical manifestations of exocrine insufficiency, as has been demonstrated by Wydmanski et al [19].

Based on our data, we suggest that TBI is detrimental for pancreatic functions, and speculate that DFO may contribute to the rapid pancreatic IO observed in these patients.

## MATERIALS AND METHODS

The study design of this single center, retrospective revision of prospectively collected data was approved by the Ethical Committee of the Institute for Research in Maternal and Child Health Burlo Garofolo of Trieste (reference no. 1105/2015), provided the patient files were anonymized. Because of the retrospective nature of this study, the requirement to obtain informed consent was waived. Written informed consent for the use of any clinical data in research was obtained for all patients at the time of admission to the Bone Marrow Transplant Unit.

### Patient selection

Inclusion criteria were as follows: age, <18 years; history of allogeneic HSCT performed between January 2012 and December 2016 for hematological or oncological diseases; MRI evaluation of iron concentrations in abdominal organs performed before transplantation and at discharge; presence of IO involving at least one of the three examined organs (liver, spleen, and bone) before transplantation; normal exocrine (serum amylase and lipase levels, and fecal elastase-1 concentration) and endocrine (insulin, c-peptide, and hemoglobin A1c levels) pancreatic functions. Exclusion criteria were refusal to participate in the study, allogeneic HSCT for diseases other than hematological or oncological diseases, normal body iron stores; pre-transplantation pancreatic IO, history of pancreatic disease, or any anatomical abnormality in pancreatic imaging.

### Transplantation procedure

Allogeneic HSCT was performed after a myeloablative standard conditioning regimen based on oral busulfan (480 mg/m^2^) or total body irradiation (TBI; 12 Gy total dose in six fractions). Myeloablative conditioning was completed with high dose leukemia-specific therapy and immunoablation therapy as described previously [30]. GVHD prophylaxis was performed with a calcineurin inhibitor, which was associated with mycophenolate mofetil in the matched unrelated and haploidentical donor groups. One haploidentical recipient had received cyclophosphamide post-transplantation.

### Chelation therapy

**Figure 3:**
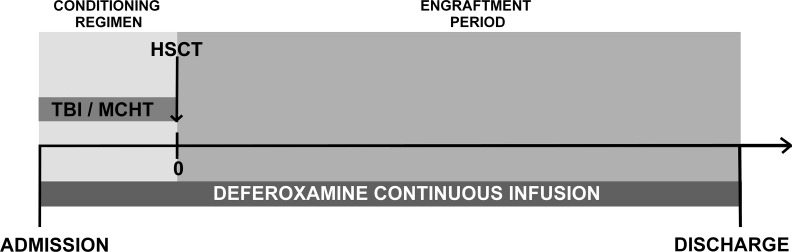
Deferoxamine chelation treatment timeline TBI, total body irradiation; MCHT, myeloablative chemotherapy; HSCT, hematopoietic stem cell transplantation.

All patients were subjected to abdominal MRI to evaluate tissue iron concentrations in before and at 1 month after transplantation. Iron concentrations were calculated in the liver (LIC), pancreas (PIC), spleen (SIC), and bone (BIC). Mean tissue iron concentrations (MTICs) were calculated for each patient based on MRI-based mean LIC, PIC, SIC, and BIC values. Iron concentrations of <40 μmol/g were classified as normal, 40–100 μmol/g was classified as mild IO, 100–200 μmol/g represented moderate IO, and >200 μmol/g was classified as severe IO. All patients with severe IO or moderate IO with a high risk of engraftment delay or transplantation-related complications because of AB0 or human leukocyte antigen mismatch or a low number of infused stem cells underwent chelation therapy with deferoxamine (30 mg/kg/day continuous intravenous infusion). The chelation therapy started on admission, together with the conditioning regimen and lasted until the discharge from Transplant Unit (Figure 3).

### Evaluation of renal and liver functions

The estimated glomerular filtration rate (eGFR) in each patient was determined according to the Schwartz formula. Based on their eGFR value, patients were grouped as children with normal renal functions (eGFR: ≥90 ml/min/1.73 m^2^), children with mild renal insufficiency (eGFR: 60–89 ml/min/1.73 m^2^), and children with moderate renal insufficiency (eGFR: 30–59 ml/min/1.73 m^2^).

Liver function assessment accounted for the presence of viruses and autoimmune hepatitis, steatosis, and heavy IO based on histological evaluation. Drug-induced liver toxicity was diagnosed by either histological or biochemical abnormalities when any other cause of liver damage had been ruled out.

### Pancreatic volume assessment

All abdominal MRIs were retrieved and reviewed to recalculate iron storage in each patient before and after HSCT. Pancreatic volume was evaluated using the DICOM image viewer OsiriX (Pixmeo SARL, CH-1233 Bernex, Switzerland). To calculate pancreatic volume, the area of the pancreas was traced manually. The image analysis software calculated the segmental volume of each pancreatic slice. Total pancreatic volume was computed as the sum of the slice volumes.

### Statistical analysis

All collected data were processed by descriptive statistical analysis to obtain the distribution and frequency of variables. Parameters were expressed as the mean ± standard deviation for continuous variables, whereas categorical variables were expressed as the absolute frequency value or percentage. Non-parametric statistics were used as appropriate, considering the size of the data sets. Analysis of variance was performed to detect differences between groups of patients. The Chi-square test was used to detect differences between groups of patients and analyze percentage data. Fisher's exact test was performed to assess the strength of association of categorical variables. The The Wilcoxon test was used to compare pre- and post-transplantation paired data. Spearman's rank correlation coefficient was used to assess the relationship between clinical parameters (continuous variables). All statistical tests were two-sided, and p<0.05 was considered as significant. The analysis was performed using Win Stat software (v.2012.1, R. Fitch) and Prism 5 for Windows (GraphPad Software, Inc.).

## Abbreviations

BIC: bone iron concentration
DFO: deferoxamine
eGFR: estimated glomerular filtration rate
GRE: gradient-recalled-echo
GVHD: graft versus host disease
HSCT: hematopoietic stem cell transplantation
IO: iron overload
LIC: liver iron concentration
MCHT: myeloablative chemotherapy
MRI: magnetic resonance imaging
MTIC: Mean tissue iron concentrations
NTBI: non–transferrin-bound iron
PIC: pancreas iron concentration
PRBC: packed red blood cell
SIC: spleen iron concentration
TBI: total body irradiation.
